# Nylon 6,6 Nonwoven Fabric Separates Oil Contaminates from Oil-in-Water Emulsions

**DOI:** 10.1371/journal.pone.0158493

**Published:** 2016-07-13

**Authors:** Ryan A. Ortega, Erin S. Carter, Albert E. Ortega

**Affiliations:** 1 Department of Biomedical Engineering, Vanderbilt University, Nashville, TN, 37235, United States of America; 2 Cerex Advanced Fabrics, Inc., Pensacola, FL, 32533, United States of America; University of Akron, UNITED STATES

## Abstract

Industrial oil spills into aquatic environments can have catastrophic environmental effects. First responders to oil spills along the coast of the Gulf of Mexico in the southern United States have used spunbond nylon fabric bags and fences to separate spilled oil and oil waste from contaminated water. Low area mass density spunbond nylon is capable of sorbing more than 16 times its mass in low viscosity crude oil and more than 26 times its mass in higher viscosity gear lube oil. Nylon bags separated more than 95% of gear lube oil contaminate from a 4.5% oil-in-water emulsion. Field testing of spunbond nylon fences by oil spill first responders has demonstrated the ability of this material to contain the oily contaminate while allowing water to flow through. We hypothesize that the effectiveness of nylon as an oil filter is due to the fact that it is both more oleophilic and more hydrophilic than other commonly used oil separation materials. The nylon traps oil droplets within the fabric or on the surface, while water droplets are free to flow through the fabric to the water on the opposite side of the fabric.

## Introduction

Accidental spills of petroleum products can have profound and far-reaching environmental effects, especially if the spilled material is dispersed in water as an oil-in-water emulsion. The Deepwater Horizon oil spill in the Gulf of Mexico in 2010, for example, released an estimated 4.9 million barrels of oil into the Gulf.[1] Although 0.77 million gallons of dispersant were applied to the oil at the wellhead in order to reduce droplet size and disperse the petroleum, much of the spilled oil reached the surface of the Gulf.[2] Approximately 1,770 km of shoreline were impacted, with hundreds of miles of beaches and marshes experiencing heavy oiling.[3] In cases of accidental spill during transport, surface run-off to watershed drainage systems can contaminate nearby bodies of water, which can then further spread the hydrocarbon contaminate. Spills are not the only mechanism by which aquatic environments can become contaminated with oil. Many industrial processes generate oil-in-water emulsions as waste products. Oily wastewater must be treated to remove oil contaminates and to mitigate the environmental impact of the oil-in-water emulsions.[4]

Mechanical recovery of petroleum contaminants on or near the water surface is the most commonly used oil spill response technique used to treat emulsified or bulk oils with a wide range of viscosities.[5,6] In addition, mechanical methods such as skimming, gravity settling, and filtration through porous membranes are commonly used techniques for treating oily wastewater.[7] Mechanical recovery methods typically utilize a natural or synthetic oleophilic fabric to contain and/or remove oil contaminates from water. These porous fabrics act as sorption media for the oil as the water passes through them. Many different materials such as superoleophilic membranes, multi-component graft polymer filters, and nanoporous graphene membranes have been investigated as next generation oil/water separation materials, but these materials have the disadvantages of high cost, low scalability, and complex synthesis.[8–11] Currently, common media used for oil containment, separation, and filtration tend to be oleophilic, non-woven fabrics produced on an industrial scale from polymer precursors; for example, the non-woven polyolefin booms used to attempt containment of the spill of the Exxon Valdez.[12]

**Fig 1:**
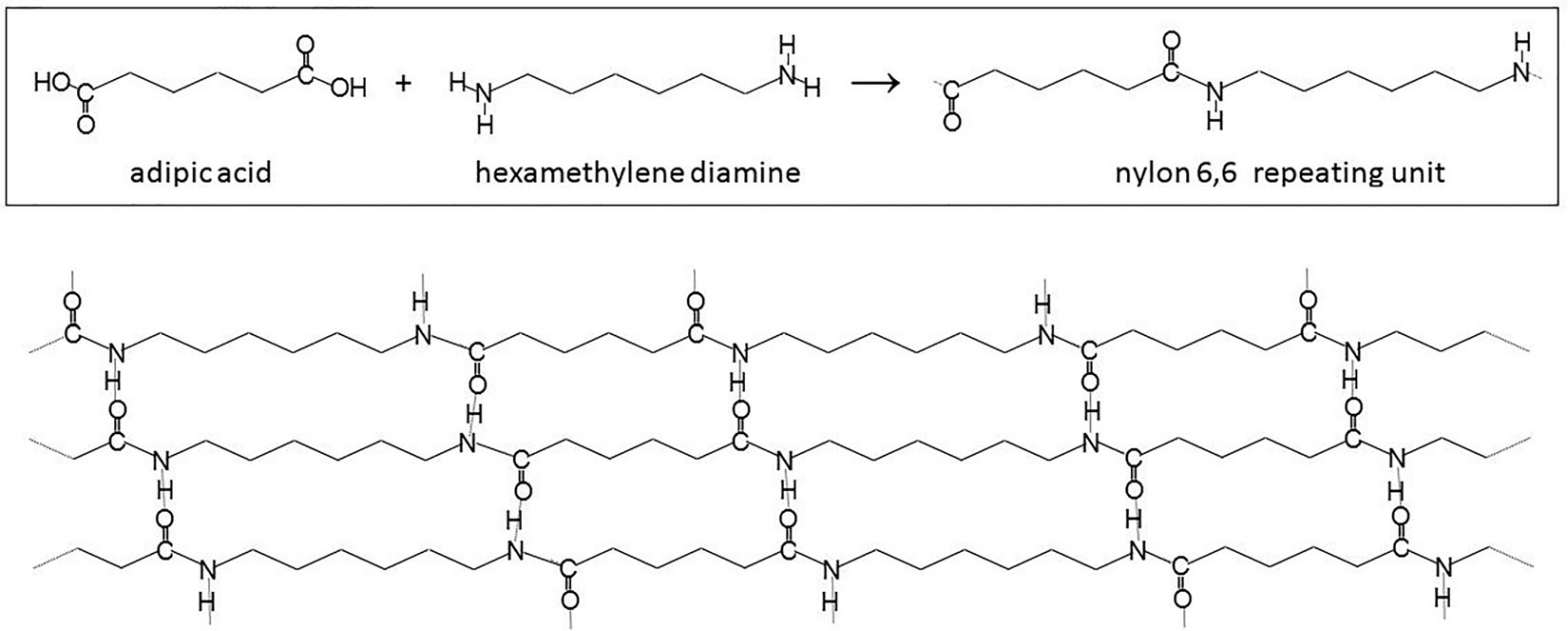
Structure of Nylon 6,6. Nylon 6,6 is a polyamide, a polymer derived from the condensation reaction of monomers containing terminal carboxylic acid (-COOH) and amine (-NH_2_) groups. The amide linkages form hydrogen bonds (indicated by grey lines) with one another in spunbond nylon fabric.

During the Deepwater Horizon oil spill, spunbond nylon 6,6 fabric was used in conjunction with polymer containment booms to remove oil contamination from Pensacola Bay.[13,14] However, there has been little investigation of the use of spunbond nylon as a material for separating oil-in-water emulsions. Nylon 6,6 is a crystalline polymer containing oleophilic hydrocarbon chains connected by hydrophilic functional amide groups (Fig 1). Nylon 6,6 is known to rapidly wet in a wide range of liquids, as evidenced by a large drop in glass transition temperature when nylon 6,6 is exposed to both polar and nonpolar solvent systems.[15] Hydrogen bonding between the polyamide chains results in high tensile strength and strong filament to filament bonding without the use of low melting copolymers or adhesives in nylon spunbond fabrics.[16] Spunbond nylon fabric made on an industrial scale in basis weights ranging from 0.3 to 4.0 ounces per square yard (osy) comprises continuous fibers which is preferred in filtration applications that require no fiber migration into the filtered medium. The fabric’s basis weight (area mass density) can be finely controlled by controlling the linear mass density and the thickness.[17]

These characteristics make spunbond nylon a promising material for oily water remediation and purification. In this study, we show that a nylon-based spunbond fabric when used in a separation system for oil-in-water emulsions effectively removes approximately 99% of high viscosity oil from an oil-in-water emulsion while absorbing/adsorbing more than 1000% of its weight in both high and low viscosity oils. We hypothesize a mechanism by which nylon spunbond fabric separates oil from water. Also, we discuss field use of nylon spunbond fabric for water remediation by the EPA and other responders to oil spill events.

## Materials and Methods

### Contact angle measurement of water and gear lube oil on nylon 6,6

Contact angles for water and gear lube oil were measured on 4.0 osy nylon spunbond fabric. Higher viscosity gear lube oil was purchased from ExxonMobil (Mobil Spartan® EP320 Gear Lube Oil) with a kinematic viscosity of 320 mm^2^s^-1^. Approximately 10 ul of either water or oil were added, dropwise, onto the nylon fabric. Images of the drops were acquired using a Dino-Lite Premier microscope at 250x magnification along with the associated DinoCapture 2.0, Version 1.4.6 software. Contact angles were measured using built in digital goniometry software.

### Oil sorption onto nylon fabric

Samples of nonwoven nylon 6,6 fabric (Type SK Oil Shark®fabric) were acquired from Cerex Advanced Fabrics, Inc. This commercially available product has a linear mass density of four denier per filament and an average pore size of 68 ± 33 μm. Crude oil samples were obtained from a private well in Louisiana, USA. The variety of crude oil acquired is designated as Light Louisiana Sweet (LLS) crude oil and has a kinematic viscosity of 3.8 mm^2^s^-1^. The higher viscosity oil used was Mobil Spartan® EP320 Gear Lube Oil, described previously. Nylon samples of different area mass density were cut into 25 mm^2^ squares (22 total nylon samples for each oil sample) and the commercially reported area mass densities were confirmed by weighing each 25 mm^2^ sample. The thickness of each sample was measured with an AMES digital thickness tester (model BG1110-1004). The sorption of the oils by the nylon samples was determined by soaking each nylon sample in either LLS crude oil or EP320 gear lube oil until saturation was achieved. The nylon samples were then removed from the oil, non-sorbed oil was allowed to drip off of the sample, and the saturated sample was weighed to determine the mass of the sorbed oil. The mass of oil sorbed was calculated as a percentage of the mass of the nylon sample. A power model regression was generated to describe the relationship between the mass of oil sorbed and the area mass density of the fabric for each type of oil.

### Separation of oil from an oil-in-water emulsion

**Fig 2:**
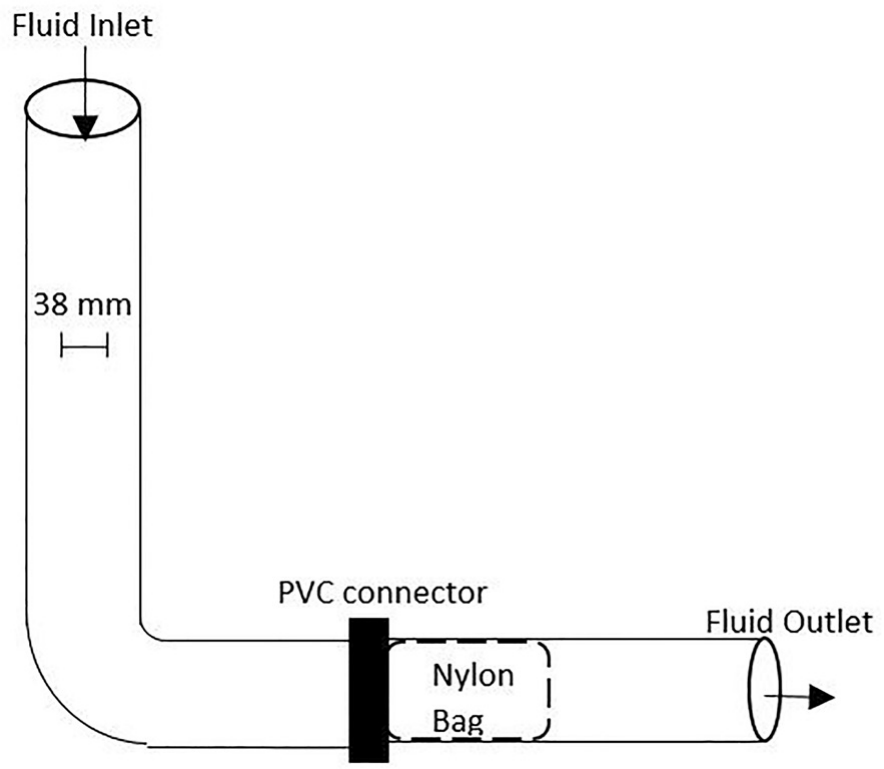
Schematic of the gravity-fed filtration rig used to test separation of the oil-in-water emulsion by nylon bags.

Oil-in-water emulsions with 4.5% oil content (vol/vol) were made by stirring seawater from the Gulf of Mexico with 320 mm^2^s^-1^ gear lube oil until well mixed. A gravity-fed filtration rig was made from 1.5 inch schedule 80 PVC pipe (internal diameter approximately 38 mm) as shown in Fig 2. Bags made from the Type SK Oil Shark® fabric and bags made from Type 60 Oil Shark® fabric were placed in the horizontal flow of the oil-in-water emulsion. The cylindrical bags had diameters of approximately 95 mm and lengths of approximately 130 mm. Each nylon bag was secured within the apparatus by inserting the mouth the bag into the threads of the PVC connector and tightening the connector around the bag. The Type 60 Oil Shark® fabric differs from the Type SK in that it has a lower linear mass density: two denier per filament. 500 ml of the oil-in-water emulsion was allowed to flow through the filtration bags. Samples of the oil-in-water emulsion were taken before and after filtration through the nylon bags. The samples were sent to TestAmerica, an environmental testing company, in Pensacola, FL, where Clean Water Act analytical method 1664A (HEM Oil & Grease) was performed to determine the concentration of gear lube oil in each sample (in ppm).[18] The total percent of oil removed from the samples was calculated using these values.

### Emergency Response Field Testing

Field testing of spunbond nylon fabric as an environmental remediation tool was carried out by first responders under the authority of the United States Environmental Protection Agency (EPA) as emergency response. In January 2014, two accidents occurred in Mississippi which resulted in the release of petroleum products into the environment and required emergency response. In the first accident, approximately 50 barrels of oil and 40 barrels of brine discharged into a wetland in Eucutta, Mississippi (Latitude: 31.756, Longitude: -88.855). In the second accident, a freight train carrying crude oil derailed in New Augusta, Mississippi (Latitude: 31.206, Longitude: -89.059) releasing 50,000 gallons of crude oil, petroleum distillate, and methanol. In both cases, nylon fabric was used by emergency first responder personnel to contain the environmental contaminates and to filter contaminated ground water. Field studies did not involve any endangered or protected species.

## Results and Discussion

### Oil sorption onto nylon

**Fig 3:**
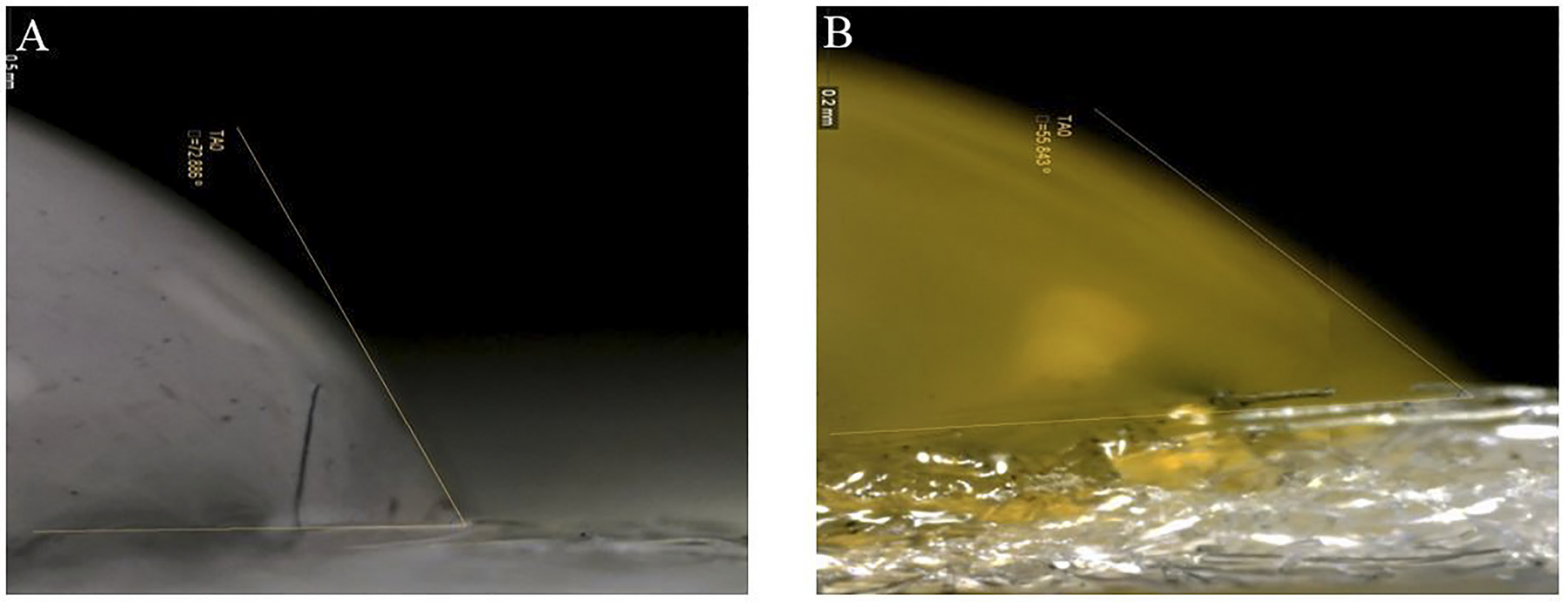
The contact angles of water and gear lube oil on nylon measured using digital goniometry. (A) The contact angle of water with 4.0 osy nylon is measured to be 73°. (B) The contact angle of gear lube oil and nylon is measured to be 56°.

Nylon 6,6 is known to be amphiphilic.[15] The amphiphilic nature of nylon is confirmed by an investigation of the contact angle of water and oil with nylon fabric. Fig 3A shows the contact angle formed by water on 4 osy nylon fabric. The contact angle measured for water on nylon is 73°, indicating that the fabric is hydrophilic.[19] Similarly, the contact angle formed by the same nylon fabric and high viscosity gear lube oil is 56°, indicating that the material is also oleophilic (Fig 3B).

**Fig 4:**
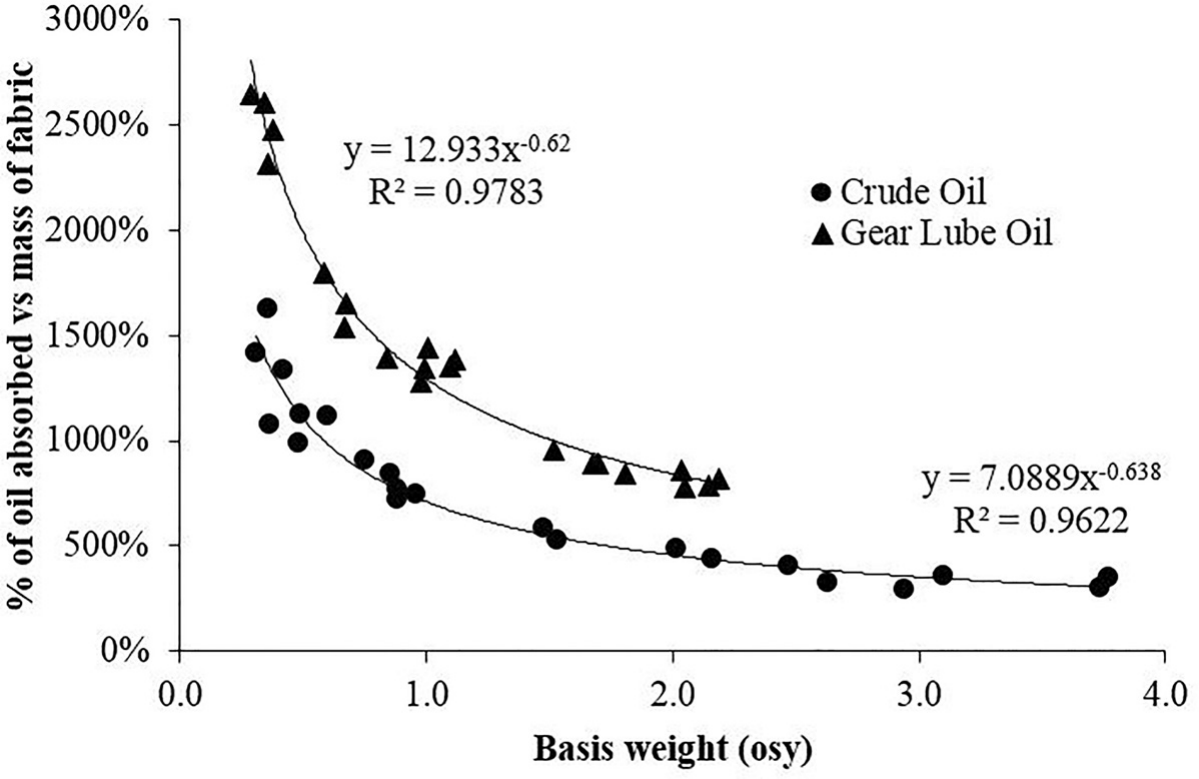
Sorption of crude oil and gear lube oil onto nylon fabric. Sorption of oils onto spunbond nylon fabric with different basis weights exhibits a power law relationship.

The spunbond nylon samples used in this study sorb oils following a power model (Fig 4). At the lowest fabric densities measured, the fabric sorbed approximately 16x its weight in crude oil and 26x its weight of the more viscous gear lube oil. Sorption of oil for both viscosities is a function of the power of the fabric’s area mass density (basis weight, osy) raised to the power of -0.638 for crude oil and -0.62 for the gear lube oil. These powers are almost identical, indicating similar sorption behavior for the nylon fabric, with differences in oil viscosity affecting the coefficient of the power function (12.93 for gear lube oil and 7.09 for crude oil). This indicates that the material properties of the nylon fabric (constant for this experiment) control the value of the exponent of the power function, and therefore the shape of the curve, while the viscosity of the oil affects the coefficient of the power function and the separation between the two curves in Fig 4. Higher basis weight fabrics have a larger mass of nylon packed into approximately the same area as lower basis weight fabrics. Though the lower basis weight fabrics have less total material, they have more void space for containing oil droplets and more available sorbent surface area due to reduced contact between the less dense nylon strands.

### Oil-in-water emulsion separation with nylon bags

Both the two denier per filament and four denier per filament nylon bags filtered more than 97% of the oil contaminates from the oil-in-water emulsion (Table 1). Nylon spunbond fabric contains oleophilic aliphatic chains which are connected by hydrophilic polyamide linkages.[20] Upon contact with water molecules, the polyamide linkages are solvated while the aliphatic chains in the nylon are available to participate in Van der Waals attractions with the non-polar oil.[16] Because of the ability of the shell hydration around each polyamide linkage to grow large enough to merge with each other in the aqueous environment, the emulsified oil initially attracted to the aliphatic chain separates from the nylon as it is repelled by the polarity of the relatively large hydration shell. The enlarging oil aggregation is held together by the surface tension of the oil which is in an aqueous environment. If the force applied by the flowing water upstream of the coalesced oily aggregate is lower than the repulsive force applied by the oleophobic, solvated polyamide linkages, the oily aggregate will be kept from flowing through the hydrated nylon barrier. The oil aggregation increases in size by coalescing oil particles out of the oily water being filtered. In an aqueous system, the water on the opposite side of the fabric represents a significant thermodynamic barrier to oil release from the nylon, resulting in oil retention in the fabric as opposed to oil release into the water on the other side.

**Table 1:** Results of the oil-in-water emulsion separation tests using nylon fabric of differing linear mass densities.

Nylon Fabric Type	Basis Weight	Oil content in original sample (ppm)	Oil content in final sample (ppm)	% Oil removed
	2	43,000	510	98.8%
Type SK Oil Shark	3	47,000	370	99.2%
	4	43,000	1100	97.4%
	2	43,000	240	99.4%
Type 60 Oil Shark	3	47,000	190	99.6%
	4	43,000	140	99.7%

**Fig 5:**
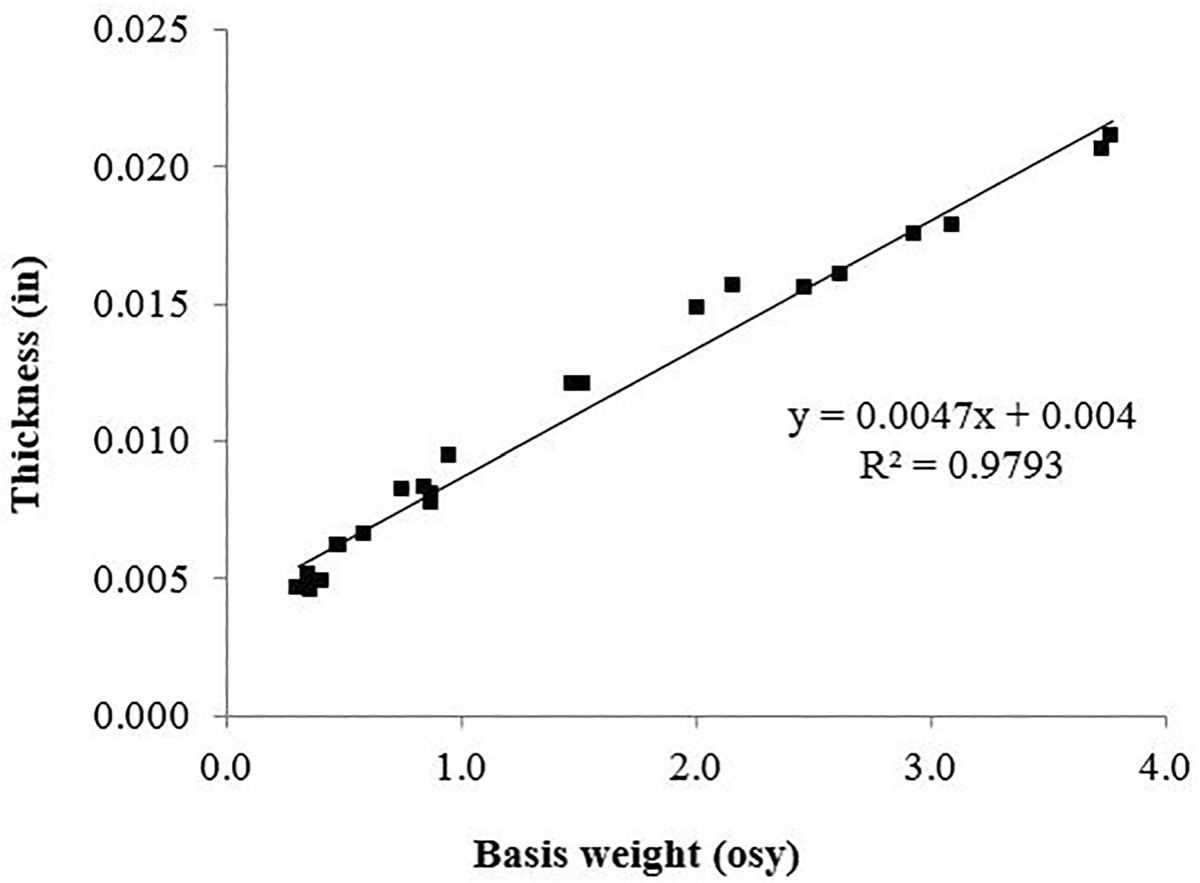
Fabric thickness scales linearly with the basis weight.

When a filtration media is placed in the flow path of an oil and water emulsion to separate the oil from the water, there will be a maximum amount of oil that the fabric will retain. The maximum oil retained is referred to as the critical oil exposure volume (COEV), or saturation volume. It is known that COEV decreases with increasing fabric thickness because fluids will take a longer time to diffuse though thicker fabrics.[21] Although the body of water on the far side of the fabric creates a significant barrier that resists oil transport through the fabric, oil droplets occasionally pass through the fabric filter. In these cases, droplets passing through thicker fabric have had more time to coalesce, resulting in the release of larger oil droplets from the fabric. Fig 5 shows that the thickness of the studied fabric increases linearly with respect to basis weight, providing evidence that the mechanism driving the oil separation efficiency is the mechanism described above, first hypothesized by Briscoe, et al.[21]

The nylon spunbond fabric also affects the stability of the interfacial distance between the surface of the fabric and the oil. Since the oil is emulsified in water, it is conjectured that the hydrophilicity of nylon spunbond fabric provides a motive force to attract the aqueous emulsion to the fabric increasing the initial rate at which the oil adheres to the fabric. The surface energy of nylon in an n-alkane/water system, 52.9 mJ/m^2^, is higher than that of polyester, 40 mJ/m^2^, or polyethylene, 23.1 mJ/m^2^, commonly used materials in sorbent booms used for oil reclamation.[22,23] This suggests that nylon is more hydrophilic than polyester or polyethylene in a crude oil-in-water system. Polyethylene behaves similarly to polypropylene and has surface tension that is near identical to polypropylene.[24] The moisture regain of nylon 6,6 at 65% relative humidity and 23°C is 4.5% which is higher than the moisture regain of polyethylene terephthalate (PET) and polypropylene at about 0.4% and about 0.05%, respectively.[25] Thus, nylon 6,6 is both hydrophilic and oleophilic. In most cases large bodies of water, lakes, rivers, and streams will not flow fast enough to provide the force needed to overcome the attractive forces within the oil and the repulsive forces of the shell of hydration surrounding the polyamide linkages. Therefore, the oil will remain upstream of the nylon spunbond fabric. This will allow execution of methods to remove the oil from the environment.

### Field testing of nylon fabric for environmental remediation following oil spills

**Fig 6:**
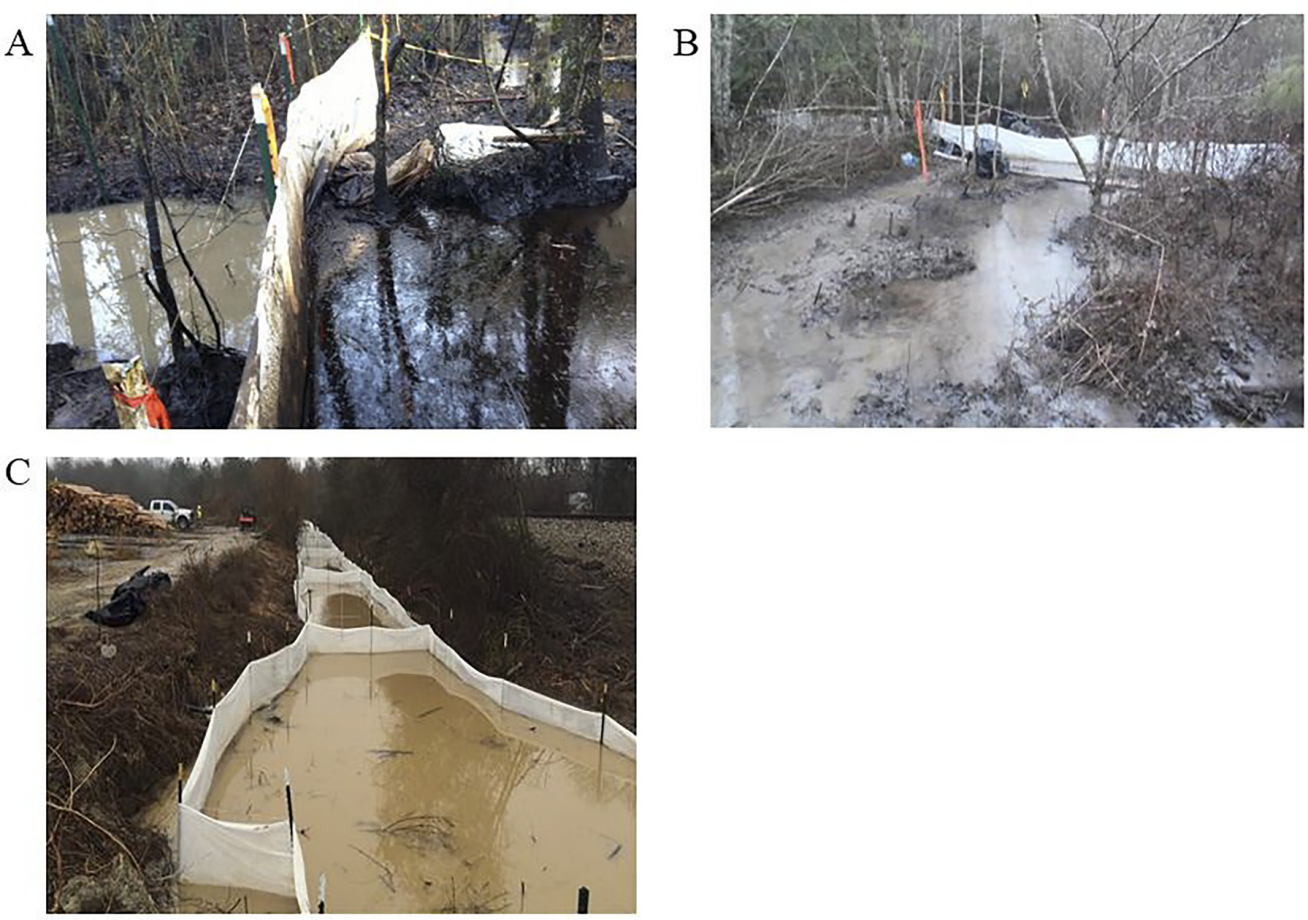
Field testing of spunbond nylon fabric used to contain oil spills. (A & B) Nylon spunbond fabric is stretched across narrow waterways contaminated with oil and brine in Eucutta, Mississippi. The fabric is fixed on both banks of the small stream and the length of nylon is sunk as deep as possible into the flowing water. (C) Nylon fabric is used to construct a series of containment cells in an oil contaminated stream in New Augusta, Mississippi. The flow of the stream carries the oil-in-water emulsion through each successive unit of the filtration system. Water is able to pass through the system of containment cells, but the oil contaminate is trapped within the nylon structure.

In January 2014, approximately 50 barrels of oil and 40 barrels of brine discharged from a flow line break that was connected to a tank battery into a wetland impacting approximately 8 acres and 8 property parcels stretched out over approximately 2200 linear feet in Eucutta, Mississippi. Fencing made with Type SK Oil Shark® nylon spunbond fabric was installed by first responders to contain the crude oil and prevent further contamination of watershed drainage systems. Crews then removed the oil sequestered by the nylon fences from the wetlands (Fig 6A and 6B).[26,27] Also in January 2014, a freight train carrying crude oil derailed in New Augusta, Mississippi. Approximately 50,000 gallons of crude oil was released along with petroleum distillate and methanol. As with the spill in Eucutta, Mississippi, fences made with Type SK Oil Shark® spunbond fabric were used to contain the petroleum distillate and prevent contamination of the groundwater. First responder teams then removed the oil from the environment (Fig 6C).[28] In these instances, the nylon fences proved effective at containing the oily contaminate, and preventing disseminated contamination of surrounding water sources.

The unique properties of nylon spunbond fabric make it an effective material to use to separate oil from water in an oil-in-water emulsion. Field testing has provided qualitative evidence that spunbond nylon can be used effectively by first responders to contain oil spills in aqueous environments. Specifically, the oleophilic and hydrophilic nature of nylon spunbond fabric provides an optimal material to separate oil from water in an oil-in-water mixture. Our experiments have determined that nylon spunbond fabric is capable of sorbing several times its mass in oil and nylon spunbond fabric of lower density separates oil from water with greater efficiency, as predicted by current models of water filtration systems utilizing polymer nonwoven fabrics. This provides first responders with a new material to remediate spilled hydrocarbons and other organic fluids such as crude oil by containing, collecting, and removing these fluids from the environment.

## Supporting Information

S1 Dataset Sorption of oils onto nylon fabric. (XLS)

S2 Dataset Fabric thickness scales with fabric basis weight. (XLS)
